# Exome sequencing reveals aberrant signalling pathways as hallmark of treatment-naive anal squamous cell carcinoma

**DOI:** 10.18632/oncotarget.23066

**Published:** 2017-12-08

**Authors:** Wulfran Cacheux, Virginie Dangles-Marie, Etienne Rouleau, Julien Lazartigues, Elodie Girard, Adrien Briaux, Pascale Mariani, Sophie Richon, Sophie Vacher, Bruno Buecher, Marion Richard-Molard, Emmanuelle Jeannot, Nicolas Servant, Fereshteh Farkhondeh, Odette Mariani, Thomas Rio-Frio, Sergio Roman-Roman, Emmanuel Mitry, Ivan Bieche, Astrid Lièvre

**Affiliations:** ^1^ Département d’oncologie médicale, Ensemble Hospitalier de l’Institut Curie, Hôpital René Huguenin, Saint-Cloud, 92210 Saint-Cloud, France; ^2^ Département de Génétique, Unité de pharmacogénomique, Ensemble Hospitalier de l’Institut Curie, 75248 Paris Cedex 05, France; ^3^ Département de Recherche Translationnelle, Centre de Recherche de l’Institut Curie, 75248 Paris Cedex 05, France; ^4^ IFR71, Faculté des sciences Pharmaceutiques et Biologiques, Université Paris Descartes, Sorbonne Paris Cité, 75270 Paris, France; ^5^ Plateforme de bioinfomatique, Centre de recherche de l’Institut Curie, 75248 Paris Cedex 05, INSERM, U900 Paris, France; ^6^ Département de chirurgie oncologique, Ensemble Hospitalier de l’Institut Curie, 75248 Paris Cedex 05, France; ^7^ Centre de recherche de l’Institut Curie, UMR144 Cell migration and invasion team, 75248 Paris Cedex 05, France; ^8^ Département d’oncologie Médicale, Ensemble Hospitalier de l’Institut Curie, 75248 Paris Cedex 05, France; ^9^ Département de radiothérapie, Ensemble Hospitalier de l’Institut Curie, Hôpital René Huguenin, 92210 Saint-Cloud, France; ^10^ Département de Pathologie, Institut Curie, 75248 Paris Cedex 05, France; ^11^ Institut Curie Genomics of Excellence (ICGex) Platform, Institut Curie, 75248 Paris Cedex 05, France; ^12^ EA 7331, Faculté des sciences Pharmaceutiques et Biologiques, Université Paris Descartes, Sorbonne Paris Cité, 75270 Paris, France; ^13^ Service des maladies de l’appareil digestif, Centre Hospitalier Universitaire de Rennes, 35033 Rennes Cedex 09, Université de Rennes 1, Faculté de Médecine, 35043 Rennes, France; ^14^ Inserm ER440-Oncogenesis, Stress and Signaling, Rue Bataille Flandres-Dunkerque, 35042 Rennes, France

**Keywords:** anal squamous cell carcinoma, whole exome sequencing, somatic mutation, copy number alteration, signalling pathway

## Abstract

Anal squamous cell carcinomas (ASCC) are rare tumours in humans. The etiological role of HPV infection is now well established but little is known about the molecular landscape and signalling pathways involved in the pathogenesis of this cancer. Here we report the results from a whole exome sequencing of a homogeneous group of 20 treatment-naive ASCC. A total of 2422 somatic single nucleotide variations (SNV) were found, with an overall moderate rate of somatic mutations per tumour (median: 105 relevant SNV per tumour) but a high mutational load in 3 tumours. The mutational signatures associated with age and APOBEC were observed in 100% and 60% of tumours respectively. The most frequently mutated genes were *PIK3CA* (25%) followed by *FBXW7* (15%), *FAT1* (15%)*,* and *TRIP12* (15%), the two last ones having never been described in ASCC. The main copy number alterations were gains of chromosome 3q (affecting *PIK3CA*) and losses of chromosome 11q (affecting *ATM)*. The combined analysis of somatic mutations and copy number alterations show that recurrent alterations of the PI3K/AKT/mTOR pathway are frequent (60%) in these tumours, as well as potentially targetable alterations of other signalling pathways that have never been described in ASCC such as chromatin remodelling (45%) and ubiquitin mediated proteolysis (35%). These results highlight the possible implication of these aberrant signalling pathways in anal carcinogenesis and suggest promising new therapeutic approaches in ASCC. The high somatic mutation burden found in some tumours, suggesting an elevated neoantigen load could also predict sensitivity of ASCC to immunotherapy.

## INTRODUCTION

Anal squamous cell carcinoma (ASCC) is a rare tumour that accounts for less than 5% of all lower gastrointestinal tract malignancies in Europe [1], but its incidence has increased in the last two decades, especially in HIV men [2, 3]. Human papilloma virus (HPV) infection, predominantly by HPV-16, is the main risk factor of these tumours [4]. Other risk factors include anoreceptive intercourse, a high lifetime number of sexual partners, chronic immunosuppression and a history of smoking.

The majority of ASCC are diagnosed at a localized or locally advanced stage, where chemoradiotherapy (CRT) is the standard of care but is associated with a local failure or recurrence in approximately 30% of the patients [5]. A salvage abdominoperineal resection is then indicated but 30 to 60% of operated patients will experience a locoregional and/or metastatic recurrence [6, 7]. In these patients with an inoperable locally advanced or metastatic disease, very few treatments are available and their effectiveness is limited. New therapeutic approaches and predictive factors of outcome are required in this context.

A better understanding of molecular markers involved in anal carcinogenesis is required for the identification of new therapeutic targets as well as prognostic and predictive biomarkers. In contrast to other squamous cell carcinomas (SCC) and HPV-related cancers for which several recent large-scale studies have respectively defined the genetic and epigenetic processes involved in oncogenesis [8], the molecular landscape of ASCC is currently not completely characterized. Previously studies have revealed frequent alterations in the PIK3CA/AKT/mTOR pathway, high levels of EGFR expression but few *KRAS* or *BRAF* mutations [9–13]. Most of these analyses were performed on a limited number of genes or in tumours previously treated by CRT. Only two exome-wide mutational studies are available: the first study of Mouw *et al.* [14] evaluated the impact of CRT on genomic evolution in ASCC recurrences samples (after initial curative CRT) and the second study of Morris KW *et al.* evaluated the genomic evolution in metastatic ASCC samples [15].

To identify potential driver genes and to better understand molecular markers involved in anal squamous carcinogenesis, we performed a whole exome sequencing (WES) analysis focused on a homogeneous cohort of 20 frozen treatment-naive ASCC patients, and identified recurrent somatic alterations and common altered signalling pathways.

## RESULTS

### Patients and tumour characteristics

From a large previous reported cohort of ASCC [10], we identified and included in the present study 20 treatment-naive and fresh frozen tumour tissues for WES analysis. Patients and tumour characteristics were summarized in Table 1. All tumours were HPV positive, with HPV16 in 19 cases and HPV6-11 in the only HIV positive patient of the series. Most of the tumours (80%) were moderate or well differentiated. All patients except one were treated by CRT or radiotherapy (RT) preceded by surgery or tumour excision in 4 of them.

**Table 1:** Clinico-pathological features of the 20 treatment-naive ASCC patients

Clinico-pathological features	Number of patients, *n*
**Total**	20
**Gender**	
Female	16
Male	4
**Concomitant HIV infection**	
Yes	1
No	19
**Tumour differentiation**	
Moderate/Well	16
Poor	4
**HPV status**	
HPV positive	20
genotype 16	19
genotype 6–11	1
HPV negative	0
**Initial tumour stage (AJCC-2010)**	
I	1
II	3
IIIA	8
IIIB	6
IV*	1
ND	1
**Initial therapy**	
Surgery (tumour excision/APR):	5
alone	1
followed by radiotherapy	3
followed by chemoradiation	1
Radiation	1
Chemoradiation	14
with concomitant 5FU-CDDP	11
with concomitant 5FU-MMC	1
with other concomitant Chemo	2

*liver metastasis

APR: abdominoperineal resection; CDDP: cisplatin; Chemo: chemotherapy; MMC: mitomycin C

### Mutational spectrum

**Figure 1:**
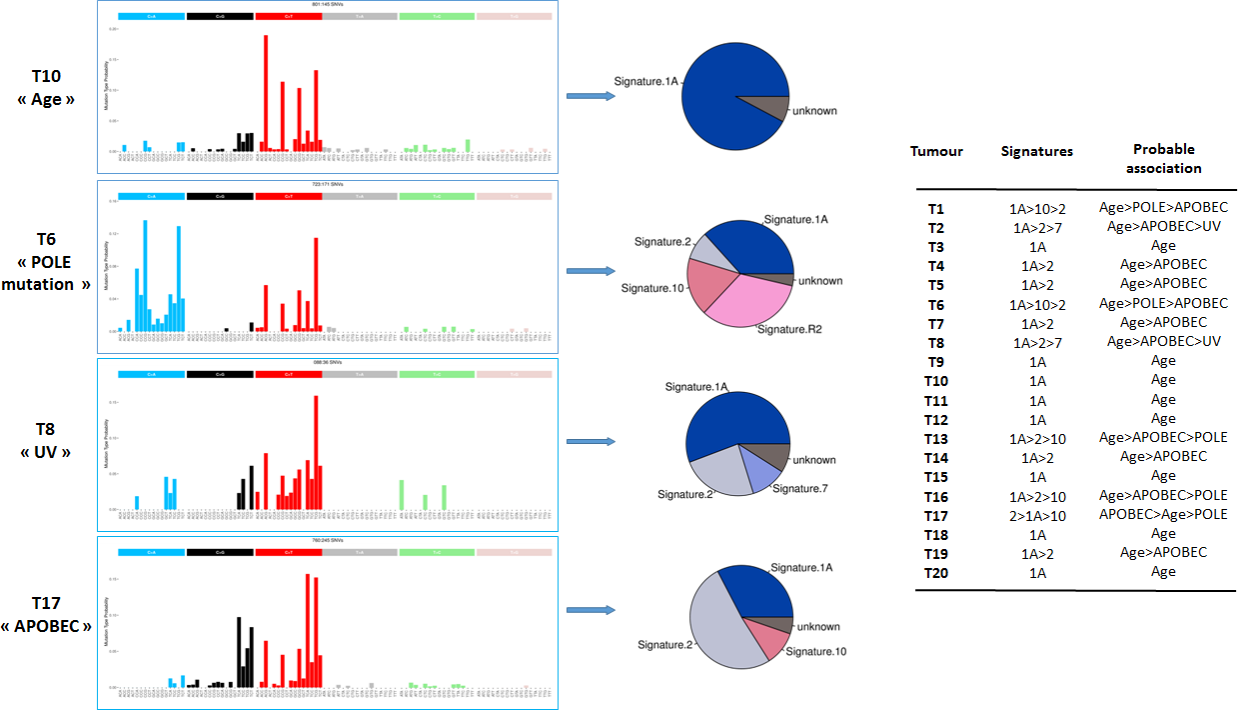
Mutational trinucleotide signatures in the series of 20 ASCCs Mutational trinucleotide signatures were determined by approaches described in the Materials and Methods section.^18^ Signature 1A (Age) is characterized by C>T in ACG, CCG, GCG or TCG; signature 2 (APOBEC) by C>T and C>G in TCA or TCT ; signature 7 (UV) by C>T in TCG; and signatures 10 (POLE mutation ) by C>T in TCG and C>A in TCT. Signature R2 is not validated.

WES of 20 tumours and matched normal samples targeted 216320 exons in 23103 genes, for a total of 50.4 Mb of captured regions. On average, 62M reads were sequenced per sample and 96.2% aligned on the reference genome, among which 31.2% were identified as PCR duplicates. Overall, the mean depth of coverage of the targeted regions was 119X and 51% of the exome was covered by more than 100X. In the total of 20 treatment-naive anal carcinoma samples, WES identified 2396 somatic variants in 2049 genes: 660 (27.3%) synonymous variants, 1492 (61.6%) missense variants, 25 in-frame indels (1.0%) and 219 protein-truncating variants (9.0%), including 38 splicing variants, 61 frameshift indels, 116 nonsense variants and 4 stoploss variants. The average number of non-synonymous mutations was 75 per tumour, ranging from 25 to 182 variants per tumour. Mutational trinucleotide signature in the 20 ASCC as determined by approaches described in the Materials and Methods section is showed in Figure 1. The signature associated with age (signature 1A) was observed in all tested ASCC. After the signature associated with age, the more frequent mutational trinucleotide signature, observed in 12 out of 20 tested ASCC, was the signature associated with APOBEC (“apolipoprotein B mRNA editing enzyme, catalytic polypeptide-like”) family (signature 2), followed by signatures associated with POLE mutations (5 cases) and UV light exposure (2 cases).

### Recurrent gene mutations

The 15 genes mutated in at least two tumours (≥10%) are described in Table 2. The majority of mutations were confirmed using Sanger analysis. *PIK3CA* mutations were found in 5 (25%) of the 20 ASCC, corresponding to the classical somatic activating hot-spot mutations described in the COSMIC database of somatic mutations in cancer [16]. The three other genes the more frequently mutated were *FBXW7* (*n =* 3), *FAT1* (*n =* 3)*,* and *TRIP12* (*n =* 3). It is noteworthy that the *FBXW7* gene is a well-known gene frequently mutated in various histological types of cancer [17–19]. The three *FBXW7* mutations (2 missenses and 1 nonsense) were all referenced as recurrent mutations in COSMIC database [16]. The *FAT1* gene has been described as a tumour suppressor gene involved in Wnt/β-catenin signalling [20] and frequently mutated in SCCs [16, 21]. *TRIP12* is an ubiquination-related gene less frequently reported as mutated in cancers. Mutations of this gene were essentially described in gastric and colorectal cancer with microsatellite instability, but also in the cervix carcinomas [16, 22]. The comparison of the observed mutation frequencies of the genes *FAT1* and *TRIP12* in various common cancers is indicated in the Supplementary Table 1. The 11 other genes mutated in 2 of the 20 tumours (10%) were *PHLDA3, MCM4, TAGAP, ASXL1, MCC, CYLD, ZNF469, ZAK, NOTCH1, MLL3* and *DDX5*.

**Table 2:** Frequently mutated genes in the series of 20 ASCCs

Gene	Chromosomal location	Number of mutated samples	%	Mutations (*n*)
*PIK3CA* NM_006218	3q26.3	5	25	MS (5) exon10:c.1624G>A:p.E542K exon10:c.1624G>A:p.E542K exon10:c.1633G>A:p.E545K exon21:c.3140A>T:p.H1047L exon21:c.3062A>G:p.Y1021C
*FBXW7* NM_033632	4q31.3	3	15	NS(1), MS(2) exon8:c.1177C>T:p.R393X exon10:c.1435C>G:p.R479G exon9:c.1322G>A:p.R441Q
*FAT1* NM_005245	4q35	3	15	NS (2), MS (1) exon2:c.1882C>T :p.R628X exon11:c.9014C>G:p.S3005X exon2:c.440C>T:p.P147L
*TRIP12* NM_004238	2q36.3	3	15	MS(1), FS(1), NS(1) exon33:c.4783C>T:p.R1595X exon37:c.5462_5469del:p.1821_1823del exon3:c.590C>T:p.S197F
*PHLDA3* NM_012396	1q31	2	10	MS(2) exon1:c.315C>A:p.F105L exon1:c.110G>A:p.G37E
*MCM4* NM_005914	8q11.2	2	10	FS(1), MS(1) exon8:c.977_978del:p.326_326del exon7:c.793G>A:p.A265T
*TAGAP* NM_054114	6q25.3	2	10	MS(1), NS (1) exon10:c.1130C>G:p.S377X exon4:c.118G>A:p.E40K
*ASXL1* NM_015338	20q11	2	10	NS(2) exon11:c.1249C>T:p.R417X exon12:c.2593G>T:p.E865X
*MCC* NM_002387	5q21	2	10	MS(1), NS(1) exon9:c.1176G>T:p.K392N exon12:c.1469C>A:p.S490X
*CYLD* NM_015247	16q12.1	2	10	MS(1), NS(1) exon11:c.1616G>A:p.C539Y exon12:c.1794C>A:p.Y598X
*ZNF469* NM_001127464	16q24	2	10	MS(1), NS(1) exon2:c.7453C>T:p.Q2485X exon2:c.4766C>T:p.P1589L
*ZAK* NM_016653	2q24.2	2	10	NS(1), MS(1) NM_133646:exon12:c.1052C>T:p.S351L NM_016653:exon13:c.1045C>T:p.Q349X
*NOTCH1* NM_017617	9q34.3	2	10	MS(2) exon23:c.3859C>T:p.R1287C exon6:c.976G>A:p.G326S
*MLL3* NM_170606	7q36.1	2	10	MS (1), NS(1) exon42:c.9638C>G:p.S3213X exon7:c.965A>T:p.H322L
*DDX5* NM_004396	17q21	2	10	MS (2) exon12:c.1251C>G:p.D417E exon5:c.496G>A:p.D166N

FS: frameshift indels, MS: missense variants, NS: nonsense variants

### Microsatellite and chromosomal instability

**Figure 2:**
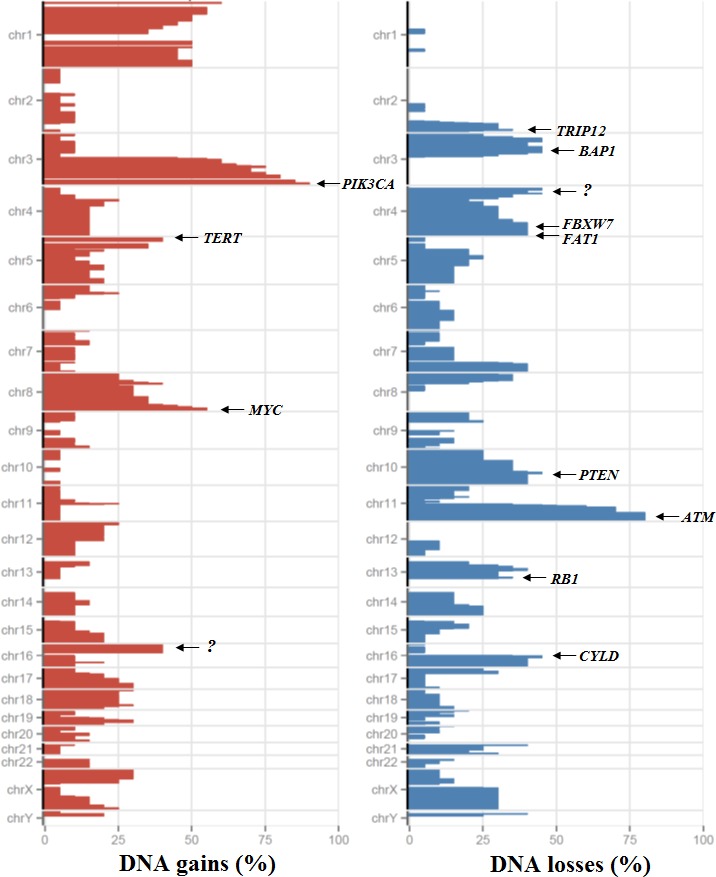
Somatic copy number alterations in the series of 20 ASCCs Frequency of copy number gains (red) and losses (blue) in *x* axis, chromosome position in *y* axis. “?” indicates the absence of well characterized driver gene.

No MSI was observed in the 20 ASCC. The Figure 2 illustrates the SCNA observed in the 20 tumours. The statistical analysis identified 6 regions with relevant gains and 8 regions with relevant losses of DNA copy number. Full arm chromosomal gains were observed in 1p (*n =* 4), 1q (*n =* 7), 3q (*n =* 7) and 8q (*n =* 3). In addition, focal gains were observed in 1p, 3q, 5p, 8q and 16p (Supplementary Table 2). The focal gain in 3q observed in 18 of 20 (90%) tumours affects the *PIK3CA* gene. *MYC* and *TERT* genes were also affected by focal gains in 8q and 5p observed in 55% and 40% of the tumours respectively (Figure 2 and Supplementary Table 2). In addition, 4 well-known oncogenes showed focal amplifications (characterized as described in Material and methods section): *DDR2* (1q23.3; *n =* 1), *CCND1* (11q13; *n =* 3), *AKT2* (19q13.2; *n =* 2) and *MDM2* (12q14.3; *n =* 1). We validated the focal status of gene amplification (size < 10Mb) for 2 tumours (focal amplification of *CCND1* for tumour T8 and focal amplifications of *DDR2, AKT2* and *MDM2* for tumour T16) using array comparative genomic hybridization (Supplementary Figure 1). Full arm chromosomal losses were observed in 3p (*n =* 3), 4p (*n =* 4), 4q (*n =* 2), 16q (*n =* 4) and Xq (*n =* 6). Focal deletions were also identified in 2q, 3p, 4p, 4q, 10q, 13q, and 16q in 35% to 45% of the tumours (Supplementary Table 2). Among them, deletions in 4q (*FAT1* and *FBXW7*) and 2q (*TRIP12*) affect genes that are frequently mutated in some cancer types, suggesting a tumour suppressor role of these ones (Table 2 and Supplementary Table 2). Allelic losses in 16q that contains the putative tumour suppressor gene *CYLD* were also observed in 9 of 20 (45%) tumours. A deletion of the *PTEN* locus in 10q was identified in 9 tumours, including the one harbouring a *PTEN* mutation. We observed biallelic inactivation (mutations + LOH) for 1 of the 3 *FAT1* mutated tumors, for 3 of the 3 *TRIP12* mutated tumors and for the 2 *CYLD* mutated tumors (Table 2). We suggested that other biallelic inactivation of *FAT1, TRIP12* and *CYLD (*as well as *PTEN)* could occur through promoter methylation of these four genes in the tumours showing only monoallelic inactivation (only LOH without somatic mutation or only somatic mutation without LOH).

Other focal deletions involve tumour suppressor genes in 3p (*BAP1, FHIT, SETD2, MLH1, TGFBR2, CTNNB1* and *PBRM1*), 13q (*RB1*) and 11q22-q25 (*ATM*) without any mutations found in these genes. In addition, 5 homozygous deletions were identified for the following loci: *EED* (11q14.2-q22.3), *CHL1* (3p26.1), *STK11* (19p13.3), *LDB1* (10q14) and *RB1* (13q14.2).

### DNA alterations in one primary tumour and its matched metastasis

**Figure 3:**
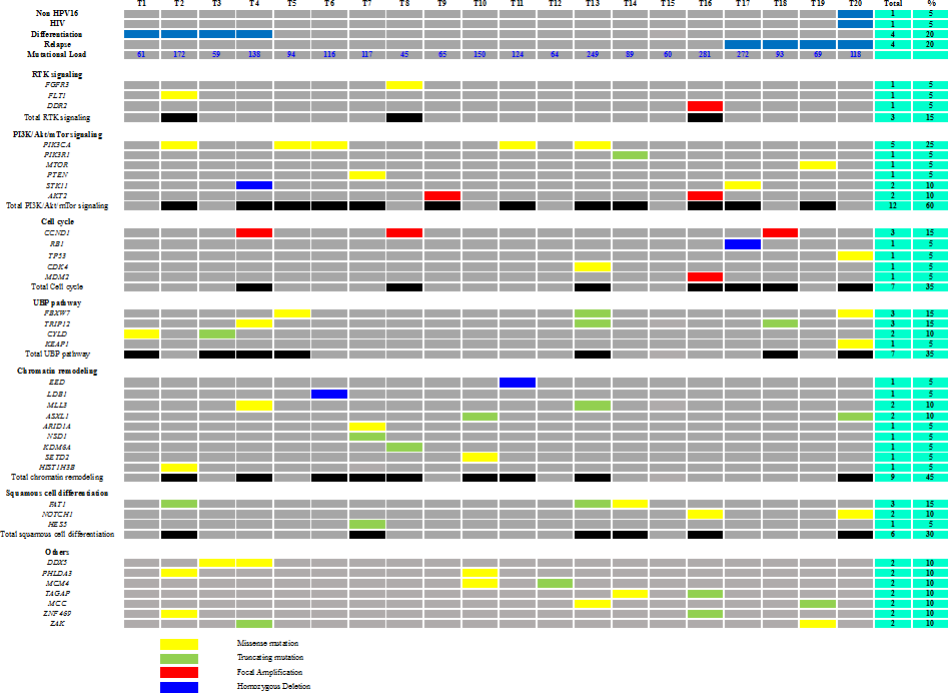
Common genetic alterations by signalling pathways in the series of 20 ASCCs

**Figure 4:**
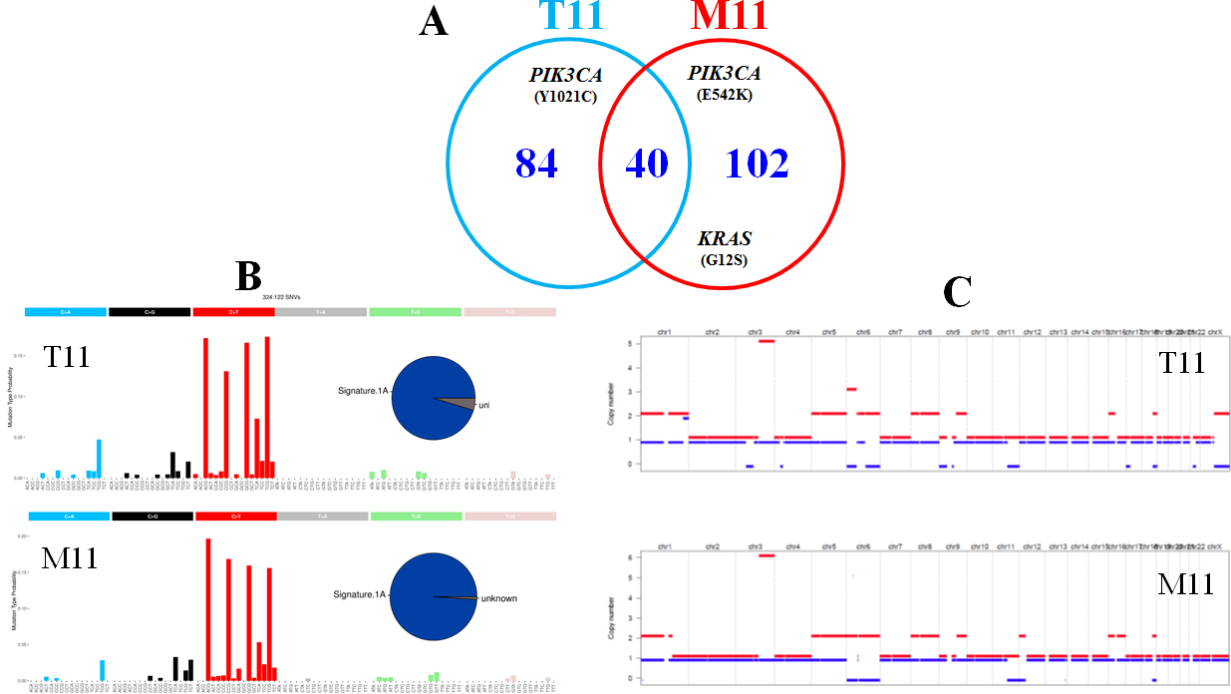
DNA alterations in one primary tumour (T11) and its matched metastasis (M11) (**A**) mutational load (number of mutations per tumour). (**B**) mutational signature as determined by approaches described in the Materials and Methods section. (**C**) Copy number analyses. The two different homologous chromosomes for each couple are showed using two different lines. Copy number gains are in red and copy number losses in blue.

For one primary tumour (T11), we performed WES in its matched pre-treated metachronous metastasis (M11). Similar somatic genomic alterations were observed between the primary tumour and the metastasis, including mutational load (124 mutations *versus* 142 mutations, with 40 mutations in common), mutational signature and SCNA profiles, with an *EED* homozygous deletion (Figure 3 and Figure 4). However, it is noteworthy that the metastasis M11 exhibited a mutation in the *KRAS* gene (G12S) that was not found in the primary tumour and a *PIK3CA* mutation (E542K) different from this observed in the primary tumour T11 (Y1021C). The mutational status (*KRAS* and *PIK3CA* genes) of the primary tumour (T11) and its matched metastasis (M11) was confirmed by Sanger analysis.

### Significant altered signalling pathways

We integrated mutation and SCNA data to characterize genomic alterations in the most frequently altered signalling pathways in SCCs [8]. Among the SCNAs, only the focal amplifications and the homozygous deletion have been considered relevant. The 5 most frequently altered pathways were the PI3K/AKT/mTOR pathway with 12 of 20 tumours (60%) harbouring focal amplifications and/or homozygous deletions and/or mutations, followed by the chromatin remodelling (45%) and ubiquitin mediated proteolysis (UBP) (35%) pathways and those involved in cell cycle regulation (35%) and squamous cell differentiation (30%) (Figure 3). No gene of the RAS/MAPK signalling pathway was altered.

Alterations in the PI3K/AKT/mTOR pathway included activating mutations of *PIK3CA* and *MTOR*, inactivating mutations (or homozygous deletions) of *PIK3R1, PTEN* and *STK11* and focal amplification of *AKT2.* All these somatic events were mutually exclusive.

The UBP pathway was altered in 7 tumours with mutations in *TRIP12, FBXW7, KEAP1* and *CYLD*. In this UBP pathway, two genes less well known to be involved in human carcinogenesis were identified: *TRIP12* and *CYLD*. These two putative tumour suppressor genes were located in chromosome 2q and 16q respectively that were frequently deleted in our series (Figure 2).

The three other frequently altered pathways in our series were the squamous cell differentiation pathway, where the totality of somatic events (*FAT1, NOTCH1* and *HES5*) were also mutually exclusive, the cell cycle regulation (*CCND1, RB1, TP53, CDK4* and *MDM2*) and the chromatin remodelling pathways (*EED, LDB1, MLL3, ASXL1, ARID1A, NSD1, KDM6A, SETD2* and *HIST1H3B*).

### Correlation between altered pathways and clinico-histological parameters

No significant association was found between the five mainly altered pathways and tumour differentiation status or recurrence (Figure 3), which might be due to the small number of samples tested.

It is noteworthy that the only HPV16 negative sample (T20) did not show any alteration of the PI3K/AKT/mTOR pathway but harboured a mutation of *TP53*, a gene rarely mutated in ASCC [10, 23].

## DISCUSSION

Genomic characterization of ASCC is crucial to better understand molecular mechanisms involved in anal carcinogenesis and identify new therapeutic targets and predictive biomarkers of response to chemoradiation. The integration of HPV DNA in the human genome is recognised as the main cause of ASCC [24], through the interaction of E6 and E7 oncoproteins with tumour suppressor genes, but other somatic genomic alterations could be involved in this process [25]. Recently 2 exome-wide mutational analysis interestingly showed a similar mutational profile in matched pre-, post-chemoradiotherapy and metastatic ASCC samples [14–15]. In order to complete the characterization of ASCC genomic landscape and avoid the identification of genetic alterations that could be induced by radiation or chemotherapy, we performed WES in a homogenous cohort of 20 treatment-naive ASCCs with corresponding complete clinico-pathological data. Our cohort is representative of the overall ASCC population, with 95% of HPV16 positive tumours and a 20% rate of tumour relapse after radiation-based therapy.

Among the most frequently mutated genes identified in this present study, *PIK3CA* and *FBXW7* have been reported consistently with similar frequencies in previous studies, mostly limited to the analysis of a panel of genes and performed on heterogeneous ASCC samples [9–13]. As *PIK3CA* and *FBXW7* genes are significantly mutated in other HPV associated squamous cell carcinomas, they could act as driver events in this histological type of cancers.

We identified recurrent somatic mutations in *FAT1* (15%) and *TRIP12* (15%) genes that have not been previously reported in ASCC. The *FAT1* gene is implicated in the squamous cell differentiation and encodes a cadherin-like protein that is able to suppress cancer cell growth *in vitro* and *in vivo* by binding ß-catenin and antagonizing its nuclear localization. Recurrent somatic variants of *FAT1* have been identified in several cancers and particularly in squamous cell carcinomas [21, 26, 27]. The inactivation of *FAT1* via mutations has been demonstrated to activate Wnt signalling, tumorigenesis and cell invasiveness [21]. The second newly identified mutated gene *TRIP12,* codes for an E3 ubiquitin-protein ligase involved in ubiquitin mediated proteolysis (UBP) pathway and regulation of DNA repair. In normal cells, TRIP12 mediates ubiquitination and degradation of the isoform p19ARF/ARF of CDKN2A, a well-known tumour suppressor required for p53/TP53 activation under oncogenic stress. The alteration of TRIP12 promotes the degradation of ARF rendering cells unprotected from oncogenic stress [28]. Few mutations of *TRIP12* have been described in cancers and in particular in HPV related SCCs as only 0.5% and 1.5% of head an neck squamous cell carcinomas (HNSCC) and cervical squamous cell carcinomas (CSCC) tested were found mutated in the COSMIC database [16]. However, it was recently demonstrated that *TRIP12* is negatively regulated by HPV/p16-related protein and that the inhibition of *TRIP12* expression*,* via repression of DNA damage repair pathway, was associated with a radiosensitization in HPV-positive HNSCC cells and xenografts. Moreover, overexpression of TRIP12 was associated with poor survival in 18 patients with HPV-positive HNSCC [29] and also be a predictive biomarker of response to chemoradiation in ASCC.

We confirmed, as previously reported [10, 12, 14, 15], frequent PI3K/AKT/mTOR pathway alterations, with at least one altered partner in 60% of ASCC samples but, additionally, our data suggest a complete mutual exclusion of the different alterations affecting this pathway, which has to be taken with caution considering the small sample size of the series. The majority of the altered partners in the PI3K/AKT/mTOR pathway were identical to those reported by Chung *et al.* [12] and concerned *PIK3CA, PIK3R1, PTEN, STK11* and *AKT2*. This result is promising because PI3K/AKT/mTOR pathway alterations have been associated with clinical response to therapies targeting this pathway in other squamous cell or HPV-associated carcinomas [30–32].

Apart from PI3K/AKT/mTOR pathway, alterations of other pathways were observed in at least 30% of our cohort and concerned specifically chromatin remodelling (45%), cell cycle regulation (35%), UBP (35%), and squamous differentiation (30%) pathways. These alterations are common in many other SCCs and have been associated with potential clinical responses to targeted therapies. An alteration of other druggable targets involved in receptor tyrosine kinase signalling pathways was also observed in 15% of ASCC, concerning particularly *FGFR3*, *DDR2* and *FLT1* genes. These data, adding to those of Chung *et al.* [12] who described a number of alterations in genes involved in DNA damage pathway, chromatin remodeling, FGFR and ERBB signalling, suggest promising new targeted therapy approaches in ASCC.

It is noteworthy that the only HPV16 negative sample (T20) did not show any alteration of the PI3K/AKTmTOR pathway but harboured a *TP53* mutation. These mutations are rare in ASCC but are correlated with HPV16 negative status, resistance to chemoradiotherapy and a poor prognosis [10, 23]. We reported a global moderate rate of somatic genetic mutations in ASCCs, which is known to correlate with the production of neoantigens [33]. This should make us consider the evaluation of immunomodulatory agents in the treatment of the ASCCs showing high rate of somatic genetic mutations: 3 out of 20 tested ASCC showed a mutational load >200 somatic mutations. It is noteworthy that these three patients did not show particular clinical characteristics, and did not show germline or somatic mutation either of the gene POLE, or of one of the genes involved in DNA mismatch repair (MMR) or in homologous recombination deficiency (HRD).

In this regard, promising results have been recently reported in metastatic ASCC with the programmed death-1 (PD-1) immune checkpoint inhibitor nivolumab, in a phase 2 study [34]. Moreover, Mouw *et al.* recently described a significant and durable response to the anti-PD-1 antibody pembrolizumab in a metastatic ASCC patient whose tumour harbored a high mutational burden and neoantigens.

Based on the classification of Alexandrov *et al.* [35], we identified in our cohort a recurrent mutational trinucleotide signature, observed in 100% of tested ASCC, which was reported to be associated with age. This result is concordant with our population of patients containing a majority of elderly females with a long-term history of HPV infection. The second more frequently signature (60%) was associated with APOBEC cytosine deaminase activity, which has recently emerged as a significant mutagenic factor in human cancer and more particularly in HPV-induced cancer [36].

The WES analysis of one primary tumour and matched metastasis (T11-M11) in our series showed similar mutational load, mutational signature and SCNA profiles but revealed a different gene mutation status between the primary tumour and the metastasis, suggesting a tumour heterogeneity as this phenomenon was already observed in samples from different tumour sites in a previous study [10].

In conclusion, the WES analysis performed in our study extends knowledge of the genomic landscape of ASCC highlights the high frequency of *PIK3CA* and *FBXW7* mutations in non pre-treated primary tumours and reports the presence of new recurrently mutated genes that have not been previously described in ASCC, such as *FAT1* and *TRIP12*. The combined analysis of somatic mutations and copy number alterations reveals the presence of recurrent alterations of the PI3K/AKT/mTOR pathway, including mutations, homozygous deletions or amplifications, in 60% of the tumours and many other altered genes involved in druggable signalling pathways. The high somatic mutation burden found in some tumours, suggesting an elevated neoantigen load could also predict sensitivity of ASCC to immunotherapy. These results are obviously limited by the small sample size of the study and need to be confirmed in a larger series but they suggest promising new therapeutic approaches and bring new insights in the treatment of ASCC.

## MATERIALS AND METHODS

### Sample collection

Human samples from ASCC and matched adjacent normal anal tissues were collected and frozen at diagnosis in the Institut Curie. The adjacent normal tissues were analysed and confirmed without dysplastic component by our referent pathologic team. All biopsy tissues were residual specimens collected after initial diagnosis (from anal tumour or peripheral lymph node tissue). No patient received previous treatment (treatment-naive tumour samples). All the tumour samples were macrodissected to achieve a maximal neoplastic cellularity (more than 50%). All adjacent control samples were confirmed to be free of tumour cells. For patients without adjacent normal adjacent tissue, a peripheral blood sample was collected to extract germline DNA. This retrospective study was reviewed and approved by the Ethics Committee of the Institut Curie (No. A10-024). According to French regulations, patients were informed of research performed with the biological specimens obtained during their treatment and did not express opposition. Clinical and biological data were collected for each patient. We also analysed an additional tumour sample corresponding to a metachronous (occurred 10 months after CRT) and distant peritoneal metastasis (M11) matched to the primary ASCC (T11), which was treated with systemic chemotherapy.

### Whole exome sequencing analysis

The WES analysis was performed on paired DNA samples (tumour and matched normal control DNA). The coding sequences from individual libraries for each sample were captured using the SureSelect version 5.0 human All Exome kit (Agilent Technologies), according to the manufacturer’s protocols. Captured DNA libraries were then sequenced using the Illumina HiSeq 2500 Genome Analyzer, yielding 200 (2 × 100) base pairs from the final library fragments. Raw data were deposited on the NCBI SRA (Sequence Read Archive) database (https://www.ncbi.nlm.nih.gov/sra), under series accession number SRP119025.

### Detection of somatic mutations

After a quality control of the FastQ data using FastQC (v0.10.1, Babraham Institute), sequenced reads were aligned to the hg19 human reference genome using Burrows-Wheeler Aligner BWA (v0.6.2, 1 mismatch allowed in the 22bp-seed, a maximum of 4 differences to the reference in the whole read and 1 alignment reported per read (Li Bioinformatics 2009) and PCR duplicates were quantified and removed using Picard Tools (v1.97, http://broadinstitute.github.io/picard). Finally, only alignment intersecting the targeted sequence (Agilent SureSelect V5) were and the overall quality of the targeted regions was assessed using BedTools (v2.17.0) (Quinlan Bioinformatics 2010).

VarScan2 (v2.3.6) was then used to call both Single Nucleotide Variation (SNVs) and small insertions/deletions (indels) predicted as present in the tumours and not in their matched normal control [37]. Only reads having a mapping quality higher than 20 and bases having a base quality higher than 17 were considered in the analysis. Somatic SNV was validated only when the following criteria were met: 1) it was identified by more than distinct pairs; 2) the number of distinct tags containing a particular mismatched base was higher than 5% of the total distinct tags in the tumour sample. The estimated neoplastic cell proportion was taken into account in the analysis. ANNOVAR (10/2013 version) was then used to annotate variants with the following databases: NCBI refGene, 1000 Genomes project frequencies, ESP project (Exome Sequencing Project) frequencies and COSMIC database [38]. Mutation function importance were predicted using SIFT, PolyPhen2 and MutationTaster. In order to prioritize the variants, only those present in the coding or splicing sites in the tumour have been considered relevant. The synonymous variants were analysed separately to identify any recurrence or impact on splicing assessed with MaxEntScan algorithm [39]. Because of the limited numbers of samples available for our study, a simple enumeration was realized to analyze the data for significantly mutated genes. Mutational trinucleotide signatures were obtained using the computational framework developed by Alexandrov *et al.* and both a homemade script and the R Package (R Core team, 2015) deconstructSigs [35, 40]. In order to best reconstruct the mutational profile of the tumours, an iterative approach was performed to determine the weights to assign to each signature. To this effect, raw trinucleotide counts were normalized by the number of times each trinucleotide context is observed in the human exome.

### Sanger sequencing validation

A total of 34 relevant somatic SNVs and indels identified by WES were verified by Sanger sequencing.

### Detection of somatic copy number alterations

From the WES analysis, somatic copy number alterations (SCNA) in the tumour compared to the normal sample were detected using the R package (R Core team, 2015) Sequenza (v2.1.0) [41]. Sequenza uses read depth as well as B-allele ratio (BAF) of variants found in both germline/tumour samples to estimate the allele-specific copy number variations and the optimal ploidy and tumour cellularity, after a GC percentage correction.

Homozygous deletion and focal amplification events were characterized using FACETS (v0.5.0) that uses default parameters and a number of 50 iterations per model to obtain convergence [42]. FACETS estimates subclonal events which allows for a more accurate detection of homozygous deletion or focal amplification by discriminating them from low cellular fraction events. The mean log2 ratio in a chromosome region between 0.30 and 1.0 was classified as genomic gain, more than 1.0 (with a size <10 Mb) as focal amplification, less than –0.30 as heterozygous deletion, and less than –1.0 (with a size <5 Mb) as homozygous deletion. Relevant genes affected by a focal gain or deletion of a chromosomal region were selected using the IntOGen database [43].

### Assessment of microsatellite instability status

Microsatellite instability (MSI) status was performed on all samples subjected to WES using the MSI Analysis System (Promega, France), which is composed of 5-mononucleotide repeats (BAT-25, BAT-26, NR-21, NR-24 and MONO-27) to detect MSI and 2-pentanucleotide repeat loci to confirm identity between normal and tumour samples, following the manufacturer’s instructions.

### Human papilloma virus genotyping

HPV status has been assessed in the Pathology Department of Institut Curie. Total DNA, isolated from formalin-fixed tissue blocks, was used for HPV typing. Real-time PCR using Sybr^®^Green and specific primers for HPV16 and 18, was performed on a 7900HT Fast Real-Time PCR System (Applied Biosystems, Foster City, CA).

## SUPPLEMENTARY MATERIALS FIGURE AND TABLES



## Abbreviations

APR: abdominoperineal resection
ASCC: anal squamous cell carcinoma
CGH: comparative genomic hybridization
CNA: copy number alteration
CSCC: cervical squamous cell carcinoma
CRT: chemoradiotherapy
HNSCC: head and neck squamous cell carcinoma
HPV: human papilloma virus
MSI: microsatellite instability
RT: radiotherapy
SCNA: somatic copy number alterations
SNV: single nucleotide variation
UBP: ubiquitin mediated proteolysis
WES: whole exome sequencing

## References

[1] Glynne-Jones R, Nilsson PJ, Aschele C, Goh V, Peiffert D, Cervantes A, Arnold D, European Society for Medical O, European Society of Surgical O, European Society of Radiological Oncology (2014). Anal cancer: ESMO-ESSO-ESTRO clinical practice guidelines for diagnosis, treatment and follow-up. Eur J Surg Oncol.

[2] Forman D, de Martel C, Lacey CJ, Soerjomataram I, Lortet-Tieulent J, Bruni L, Vignat J, Ferlay J, Bray F, Plummer M, Franceschi S (2012). Global burden of human papillomavirus and related diseases. Vaccine.

[3] Piketty C, Selinger-Leneman H, Bouvier AM, Belot A, Mary-Krause M, Duvivier C, Bonmarchand M, Abramowitz L, Costagliola D, Grabar S (2012). Incidence of HIV-related anal cancer remains increased despite long-term combined antiretroviral treatment: results from the french hospital database on HIV. J Clin Oncol.

[4] Baricevic I, He X, Chakrabarty B, Oliver AW, Bailey C, Summers J, Hampson L, Hampson I, Gilbert DC, Renehan AG (2015). High-sensitivity human papilloma virus genotyping reveals near universal positivity in anal squamous cell carcinoma: different implications for vaccine prevention and prognosis. Eur J Cancer.

[5] James RD, Glynne-Jones R, Meadows HM, Cunningham D, Myint AS, Saunders MP, Maughan T, McDonald A, Essapen S, Leslie M, Falk S, Wilson C, Gollins S (2013). Mitomycin or cisplatin chemoradiation with or without maintenance chemotherapy for treatment of squamous-cell carcinoma of the anus (ACT II): a randomised, phase 3, open-label, 2 x 2 factorial trial. Lancet Oncol.

[6] Lefevre JH, Corte H, Tiret E, Boccara D, Chaouat M, Touboul E, Svrcek M, Lefrancois M, Shields C, Parc Y (2012). Abdominoperineal resection for squamous cell anal carcinoma: survival and risk factors for recurrence. Ann Surg Oncol.

[7] Mariani P, Ghanneme A, De la Rochefordiere A, Girodet J, Falcou MC, Salmon RJ (2008). Abdominoperineal resection for anal cancer. Dis Colon Rectum.

[8] Dotto GP, Rustgi AK (2016). Squamous Cell Cancers: A Unified Perspective on Biology and Genetics. Cancer Cell.

[9] Bernardi MP, Ngan SY, Michael M, Lynch AC, Heriot AG, Ramsay RG, Phillips WA (2015). Molecular biology of anal squamous cell carcinoma: implications for future research and clinical intervention. Lancet Oncol.

[10] Cacheux W, Rouleau E, Briaux A, Tsantoulis P, Mariani P, Richard-Molard M, Buecher B, Dangles-Marie V, Richon S, Lazartigues J, Jeannot E, Farkhondeh F, Sastre-Garau X (2016). Mutational analysis of anal cancers demonstrates frequent PIK3CA mutations associated with poor outcome after salvage abdominoperineal resection. Br J Cancer.

[11] Casadei Gardini A, Capelli L, Ulivi P, Giannini M, Freier E, Tamberi S, Scarpi E, Passardi A, Zoli W, Ragazzini A, Amadori D, Frassineti GL (2014). KRAS, BRAF and PIK3CA status in squamous cell anal carcinoma (SCAC). PLoS One.

[12] Chung JH, Sanford E, Johnson A, Klempner SJ, Schrock AB, Palma NA, Erlich RL, Frampton GM, Chalmers ZR, Vergilio J, Rubinson DA, Sun JX, Chmielecki J (2016). Comprehensive genomic profiling of anal squamous cell carcinoma reveals distinct genomically defined classes. Ann Oncol.

[13] Smaglo BG, Tesfaye A, Halfdanarson TR, Meyer JE, Wang J, Gatalica Z, Reddy S, Arguello D, Boland PM (2015). Comprehensive multiplatform biomarker analysis of 199 anal squamous cell carcinomas. Oncotarget.

[14] Mouw KW, Cleary JM, Reardon B, Pike J, Braunstein LZ, Kim J, Amin-Mansour A, Miao D, Damish A, Chin J, Ott PA, Fuchs CS, Martin NE (2017). Genomic Evolution after Chemoradiotherapy in Anal Squamous Cell Carcinoma. Clin Cancer Res.

[15] Morris VK, Rao X, Pickering C, Foo WC, Rashid A, Eterovic K, Kim T, Chen K, Wang J, Shaw K, Eng C (2017 Aug 7). Comprehensive Genomic Profiling of Metastatic Squamous Cell Carcinoma of the Anal Canal. Mol Cancer Res.

[16] Forbes SA, Beare D, Boutselakis H, Bamford S, Bindal N, Tate J, Cole CG, Ward S, Dawson E, Ponting L, Stefancsik R, Harsha B, Kok CY (2017). COSMIC: somatic cancer genetics at high-resolution. Nucleic Acids Res.

[17] Cancer Genome Atlas N (2012). Comprehensive molecular characterization of human colon and rectal cancer. Nature.

[18] Laforest A, Aparicio T, Zaanan A, Silva FP, Didelot A, Desbeaux A, Le Corre D, Benhaim L, Pallier K, Aust D, Pistorius S, Blons H, Svrcek M (2014). ERBB2 gene as a potential therapeutic target in small bowel adenocarcinoma. Eur J Cancer.

[19] Ojesina AI, Lichtenstein L, Freeman SS, Pedamallu CS, Imaz-Rosshandler I, Pugh TJ, Cherniack AD, Ambrogio L, Cibulskis K, Bertelsen B, Romero-Cordoba S, Trevino V, Vazquez-Santillan K (2014). Landscape of genomic alterations in cervical carcinomas. Nature.

[20] Morris LG, Kaufman AM, Gong Y, Ramaswami D, Walsh LA, Turcan S, Eng S, Kannan K, Zou Y, Peng L, Banuchi VE, Paty P, Zeng Z (2013). Recurrent somatic mutation of FAT1 in multiple human cancers leads to aberrant Wnt activation. Nat Genet.

[21] Cancer Genome Atlas N (2015). Comprehensive genomic characterization of head and neck squamous cell carcinomas. Nature.

[22] Yoo NJ, Park SW, Lee SH (2011). Frameshift mutations of ubiquitination-related genes HERC2, HERC3, TRIP12, UBE2Q1 and UBE4B in gastric and colorectal carcinomas with microsatellite instability. Pathology.

[23] Meulendijks D, Tomasoa NB, Dewit L, Smits PH, Bakker R, van Velthuysen ML, Rosenberg EH, Beijnen JH, Schellens JH, Cats A (2015). HPV-negative squamous cell carcinoma of the anal canal is unresponsive to standard treatment and frequently carries disruptive mutations in TP53. Br J Cancer.

[24] zur Hausen H (2000). Papillomaviruses causing cancer: evasion from host-cell control in early events in carcinogenesis. J Natl Cancer Inst.

[25] Thomas M, Pim D, Banks L (1999). The role of the E6-p53 interaction in the molecular pathogenesis of HPV. Oncogene.

[26] Gao YB, Chen ZL, Li JG, Hu XD, Shi XJ, Sun ZM, Zhang F, Zhao ZR, Li ZT, Liu ZY, Zhao YD, Sun J, Zhou CC (2014). Genetic landscape of esophageal squamous cell carcinoma. Nat Genet.

[27] Pickering CR, Zhou JH, Lee JJ, Drummond JA, Peng SA, Saade RE, Tsai KY, Curry JL, Tetzlaff MT, Lai SY, Yu J, Muzny DM, Doddapaneni H (2014). Mutational landscape of aggressive cutaneous squamous cell carcinoma. Clin Cancer Res.

[28] Collado M, Serrano M (2010). The TRIP from ULF to ARF. Cancer Cell.

[29] Wang L, Zhang P, Molkentine DP, Chen C, Molkentine JM, Piao H, Raju U, Zhang J, Valdecanas DR, Tailor RC, Thames HD, Buchholz TA, Chen J (2016). TRIP12 as a mediator of human papillomavirus/p16-related radiation enhancement effects. Oncogene.

[30] Dolly SO, Wagner AJ, Bendell JC, Kindler HL, Krug LM, Seiwert TY, Zauderer MG, Lolkema MP, Apt D, Yeh RF, Fredrickson JO, Spoerke JM, Koeppen H (2016). Phase I Study of Apitolisib (GDC-0980), Dual Phosphatidylinositol-3-Kinase and Mammalian Target of Rapamycin Kinase Inhibitor, in Patients with Advanced Solid Tumors. Clin Cancer Res.

[31] Hou MM, Liu X, Wheler J, Naing A, Hong D, Coleman RL, Tsimberidou A, Janku F, Zinner R, Lu K, Kurzrock R, Fu S (2014). Targeted PI3K/AKT/mTOR therapy for metastatic carcinomas of the cervix: A phase I clinical experience. Oncotarget.

[32] Janku F, Hong DS, Fu S, Piha-Paul SA, Naing A, Falchook GS, Tsimberidou AM, Stepanek VM, Moulder SL, Lee JJ, Luthra R, Zinner RG, Broaddus RR (2014). Assessing PIK3CA and PTEN in early-phase trials with PI3K/AKT/mTOR inhibitors. Cell Rep.

[33] Snyder A, Makarov V, Merghoub T, Yuan J, Zaretsky JM, Desrichard A, Walsh LA, Postow MA, Wong P, Ho TS, Hollmann TJ, Bruggeman C, Kannan K (2014). Genetic basis for clinical response to CTLA-4 blockade in melanoma. N Engl J Med.

[34] Morris VK, Salem ME, Nimeiri H, Iqbal S, Singh P, Ciombor K, Polite B, Deming D, Chan E, Wade JL, Xiao L, Bekaii-Saab T, Vence L (2017). Nivolumab for previously treated unresectable metastatic anal cancer (NCI9673): a multicentre, single-arm, phase 2 study. Lancet Oncol.

[35] Alexandrov LB, Nik-Zainal S, Wedge DC, Aparicio SA, Behjati S, Biankin AV, Bignell GR, Bolli N, Borg A, Borresen-Dale AL, Boyault S, Burkhardt B, Butler AP (2013). Signatures of mutational processes in human cancer. Nature.

[36] Henderson S, Chakravarthy A, Su X, Boshoff C, Fenton TR (2014). APOBEC-mediated cytosine deamination links PIK3CA helical domain mutations to human papillomavirus-driven tumor development. Cell Rep.

[37] Koboldt DC, Zhang Q, Larson DE, Shen D, McLellan MD, Lin L, Miller CA, Mardis ER, Ding L, Wilson RK (2012). VarScan 2: somatic mutation and copy number alteration discovery in cancer by exome sequencing. Genome Res.

[38] Wang K, Li M, Hakonarson H (2010). ANNOVAR: functional annotation of genetic variants from high-throughput sequencing data. Nucleic Acids Res.

[39] Yeo G, Burge CB (2004). Maximum entropy modeling of short sequence motifs with applications to RNA splicing signals. J Comput Biol.

[40] Rosenthal R, McGranahan N, Herrero J, Taylor BS, Swanton C (2016). DeconstructSigs: delineating mutational processes in single tumors distinguishes DNA repair deficiencies and patterns of carcinoma evolution. Genome Biol.

[41] Favero F, Joshi T, Marquard AM, Birkbak NJ, Krzystanek M, Li Q, Szallasi Z, Eklund AC (2015). Sequenza: allele-specific copy number and mutation profiles from tumor sequencing data. Ann Oncol.

[42] Shen R, Seshan VE (2016). FACETS: allele-specific copy number and clonal heterogeneity analysis tool for high-throughput DNA sequencing. Nucleic Acids Res.

[43] Gonzalez-Perez A, Perez-Llamas C, Deu-Pons J, Tamborero D, Schroeder MP, Jene-Sanz A, Santos A, Lopez-Bigas N (2013). IntOGen-mutations identifies cancer drivers across tumor types. Nat Methods.

