# Supplementary material for: Exome sequencing reveals aberrant signalling pathways as hallmark of treatment-naive anal squamous cell carcinoma
Source: Oncotarget. 2017 Dec 8;9(1):464–76. doi: 10.18632/oncotarget.23066 (PMC5787481; doi:10.18632/oncotarget.23066)
Supplement: Supplementary file 1 [file oncotarget-09-464-s001.pdf]

Exome sequencing reveals aberrant signalling pathways as hallmark of treatment-naive anal squamous cell carcinoma

SUPPLEMENTARY MATERIALS

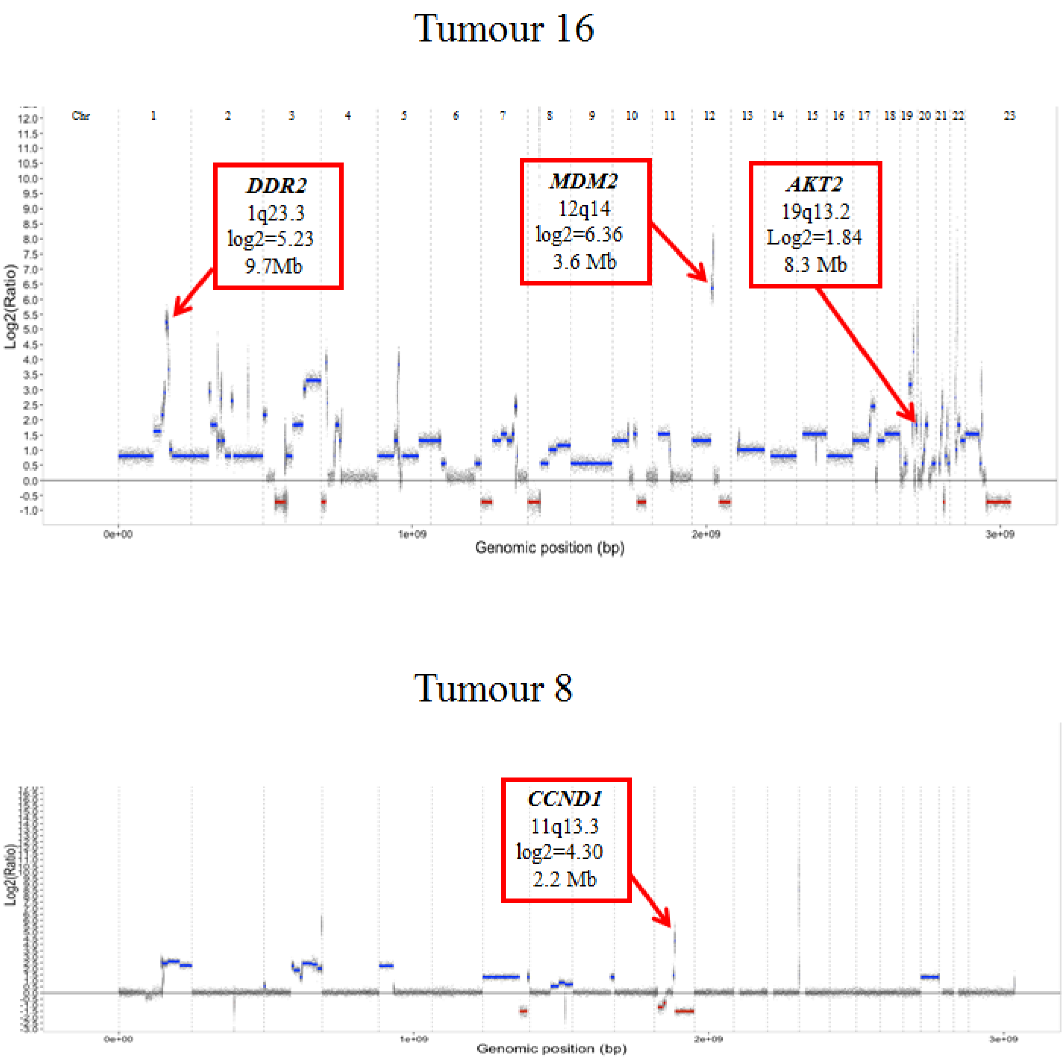

Supplementary Figure 1: Validation of focal amplifications (size < 10 Mb) using array comparative genomic hybridization.

Supplementary Table 1: Frequencies of inactivating mutations of *FAT1* and *TRIP12* in various other common cancers as compared to ASCC*

Cancers	*FAT1* % of altered tumours	*TRIP12* % of altered tumours
**ASCC (present study)**	**15 (3/20)**	**15 (3/20)**
BRCA	1.2 (6/507)	0.6 (3/507)
LUAD	11 (25/230)	3 (7/230)
LUSC	15 (25/178)	6 (11/178)
UCEC	16 (40/248)	6 (16/248)
GBM	1 (3/291)	0.3 (1/291)
HNSC	23 (64/279)	2.5 (7/279)
COAD	6 (13/224)	5 (12/224)
BLCA	12 (15/130)	4 (5/130)
KIRC	1.7 (7/424)	0.7 (3/424)
OV	3 (11/316)	1.3 (4/316)
CESC	5 (10/194)	3 (6/194)

*frequencies obtained from http://www.cbioportal.org

(ASCC) anal squamous cell carcinoma, (BRCA) breast adenocarcinoma, (LUAD) lung adenocarcinoma, (LUSC) lung squamous cell carcinoma, (UCEC) uterine corpus endometrial carcinoma, (GBM) glioblastoma multiforme, (HNSC) head and neck squamous cell carcinoma, (COAD) colon carcinoma, (BLCA) bladder urothelial carcinoma, (KIRC) kidney renal clear cell carcinoma, (OV) ovarian serous carcinoma, and (CESC) cervical squamous cell carcinoma.

Supplementary Table 2: Focal gained and deleted chromosomal regions in the series of 20 ASCCs

Chromosomal location	Genomic location	Size (Mb)	% of altered tumours	Relevant genes*
**Gain**				
1p	900506–3774138	2,8	55 (11/20)	*HES5, SIK*
3q	169643862–197432063	27,8	90 (18/20)	***PIK3CA,****TP63, TBL1XR1, FXR1, EIF4G1, EIF4A2, DLG1*
5p	678063–24509643	23,8	40 (8/20)	***TERT****, CTNND2*
8q	129021180–146076708	17	55 (11/20)	***MYC****, NDRG1*
16p	4016677–33961370	29,9	40 (8/20)	FUS, MYH11, TAOK2, CIITA
**Deletion**				
2q	209007560–233757697	24,7	35 (7/20)	***TRIP12,*** *ATIC, FN1, EPHA4, ACSL3, CUL3*
3p	21462755–73523768	52	45 (9/20)	***SETD2, FHIT, BAP1, MLH1, PBRM1, TGFBR2, CTNNB1***
4p	155625–31116375	30,9	45 (9/20)	*WHSC1, DHX15*
4q	142557749–191003549	48,4	40 (8/20)	***FBXW7, FAT1,*** *IRF2, FRG1*
10q	87379812–91352966	3,9	45 (9/20)	***PTEN***
11q	82644905–134202036	51,5	80 (16/20)	***ATM****, MED17, CASP1, KMT2A, HSPA8, ACAD8*
13q	36383265–51603768	15,2	40 (8/20)	***RB1***
16q	47347781–70711843	23,4	45 (9/20)	***CYLD, CDH1****, CHD9, NUP93, CNOT1, CBFB, CTCF*

*relevant genes using the IntOGen database[22]
